# How to Make the Skin Contact Area Controllable by Optical Calibration in Wearable Tactile Displays of Softness

**DOI:** 10.3390/s24206770

**Published:** 2024-10-21

**Authors:** Gabriele Frediani, Federico Carpi

**Affiliations:** 1 Biomedical Engineering Unit, Department of Industrial Engineering, University of Florence, 50121 Florence, Italy; gabriele.frediani@unifi.it; 2 IRCCS Fondazione don Carlo Gnocchi ONLUS, 50143 Florence, Italy

**Keywords:** wearable, tactile, touch, haptic, contact area, softness, virtual reality, calibration, control, optical

## Abstract

Virtual reality systems may benefit from wearable (fingertip-mounted) haptic displays capable of rendering the softness of virtual objects. According to neurophysiological evidence, the easiest reliable way to render a virtual softness is to generate purely tactile (as opposed to kinaesthetic) feedback to be delivered via a finger-pulp-interfaced deformable surface. Moreover, it is necessary to control not only the skin indentation depth by applying quasi-static (non-vibratory) contact pressures, but also the skin contact area. This is typically impossible with available devices, even with those that can vary the contact area, because the latter cannot be controlled due to the complexity of sensing it at high resolutions. This causes indetermination on an important tactile cue to render softness. Here, we present a technology that allows the contact area to be open-loop controlled via personalised optical calibrations. We demonstrate the solution on a modified, pneumatic wearable tactile display of softness previously described by us, consisting of a small chamber containing a transparent membrane inflated against the finger pulp. A window on the device allowed for monitoring the skin contact area with a camera from an external unit to generate a calibration curve by processing photos of the skin membrane interface at different pressures. The solution was validated by comparisons with an ink-stain-based method. Moreover, to avoid manual calibrations, a preliminary automated procedure was developed. This calibration strategy may be applied also to other kinds of displays where finger pulps are in contact with transparent deformable structures.

## 1. Motivation: Displaying Softness Requires Controlling the Fingertip Contact Area

Physically rendering the softness of computer-generated objects is a need for various kinds of virtual or augmented reality systems. Indeed, the possibility of using the sense of touch while probing the softness of a virtual object can be desirable for a diversity of applications, such as simulators for medical training [1], tele-operation systems [2], computer-aided design [3], 3D model exploration [4] and tele-presence systems for social interactions [5]. Such applications require realistic mimicking of the sensation generated by the indentation of a soft object with a fingertip. This need can be addressed with haptic displays, as human–machine interfaces that should provide users with ideally both kinds of information that synergistically encode our perception of softness: tactile feedback and kinaesthetic feedback.

Furthermore, several applications require interfaces that allow users to freely move their hands while performing a virtual or augmented reality task. This implies the need for wearable devices, sufficiently small, light and comfortable to be arranged on fingertips [6]. In such scenarios, the tactile perception task is accomplished in a so-called ‘passive’ mode, where the fingertip does not move relative to the device.

Providing a wearable interface with both tactile feedback and kinaesthetic feedback capabilities, while preserving comfort, compact size and ease of use, is still a challenge today. This includes the need for ensuring proper functionality of the system as a whole, while also ensuring that the wearable device does not hamper the tracking of the hand/fingers motions by external infrared cameras, as typically used in virtual reality setups.

However, the inherent complexity of such conceptualized haptic interfaces can be reduced, by considering that Srinivasan and LaMotte have demonstrated that the softness of objects with a deformable surface (and not just compliant with a rigid surface) can sufficiently be perceived using tactile feedback alone (whilst kinaesthetic feedback alone is insufficient) [7]. This evidence has been explained as due to the fact that, for any applied force, the object’s softness causes a deformation of the fingertip’s skin, such that differences in softness can be sufficiently detected, relying on cutaneous mechanoreceptors only [7]. Consistently, later investigations have shown how the perception of softness is mediated by variations of the contact area between fingertip and object [8,9,10], such that the change in contact area has been proposed as a new proprioceptive cue [11].

Designing such wearable tactile displays in a way that ensures realistic tactile feedback is not straightforward, as the role and interplay of different tactile cues involved in the perception of softness is still a matter of discussion [12]. Indeed, Dhong et al. have shown that, besides the contact area, another stimulus that independently contributes to the perception of softness is the indentation depth [13]. Therefore, any tactile display that exclusively controls either the contact area or the indentation depth is expected to be perceptually less performing than any device that could control both of them [13].

These considerations suggest that the easiest strategy to render the softness of virtual objects via wearable haptic displays is to use actuators that are interfaced to the skin via a deformable surface and that generate purely tactile feedback. To this end, the actuators should not only vary the skin indentation depth by applying quasi-static (non-vibratory) contact pressures, but also ensure controllability of the skin contact area. We define here such actuators as wearable (fingertip-mounted) tactile displays of softness.

## 2. State of the Art: Wearable Tactile Displays of Softness and the Problem of Contact Area Sensing

Rather few actuation technologies are currently available to develop such wearable devices with compact size, low weight and user comfort. A possible strategy is to employ electrical motors that move flexible/stretchable polymer membranes or fabrics in contact with the skin; although this type of approach has been shown to be effective, it is limited by the bulkiness and heaviness of the required mechanisms [14,15].

An alternative strategy that avoids electrical motors and transmission mechanisms exploits soft elastomeric membranes that contact the skin and can be deformed in different ways, using either dielectric elastomer (DE) actuation, electrostatic actuation or pneumatic actuation. DE actuators typically consist of elastomeric membranes with various shapes that are electrically deformed [16]; although they have been used to demonstrate a variety of non-vibratory fingertip displays [17,18,19,20,21], they are limited by the need for high driving voltages. This drawback similarly limits electrostatic actuators [22], which, like DE actuators, are also typically challenged by the high active deformations that are made possible by pneumatic actuators.

**Figure 1:**
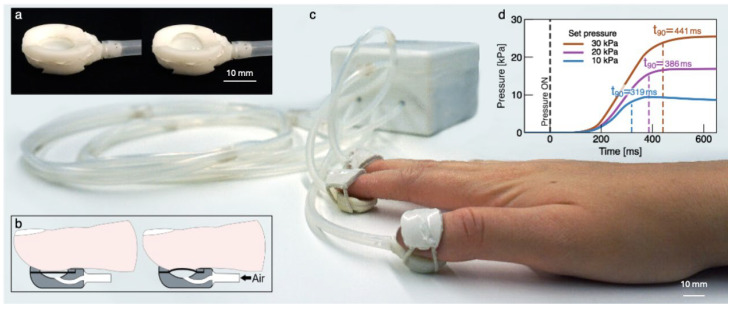
The wearable pneumatic tactile display of softness: (**a**) images of a prototype sample, showing the deformation of the membrane upon pressurization; (**b**) schematic drawings of the principle of operation, showing that, upon pressurisation, the membrane indents the finger pulp and increases their contact area, so as to provide two essential tactile stimuli for the perception of softness; (**c**) image of the complete system, where three tactile displays are independently controlled by an electropneumatic unit; (**d**) tactile pressure rise as a function of time, following the onset of the pressure stimulus (‘pressure ON’), at 10, 20 and 30 kPa.

From a general standpoint, pneumatic actuation offers a unique combination of large deformations, high forces and electrical safety. Pneumatic tactile displays have been demonstrated by using focused air jets [23,24] and pressurised chambers [25,26,27,28,29,30]. In the former, which are not wearable, the perceptual experience’s realism is limited by the lack of a soft interface with the skin. Conversely, the use of pressurised soft chambers is in our opinion more promising. Accordingly, we have recently presented a wearable pneumatic tactile display of softness [31], consisting of a small inflatable chamber arranged at the fingertip. In particular, the chamber has a plastic structure, which radially constrains a circular elastomeric membrane. As the chamber is pressurised, the membrane pushes against the finger pulp, producing an indentation of the skin and an increase in the contact area between them, as shown in Figure 1.

The perceptual performance of such tactile display technology has been studied with preliminary psychophysical [31] and electroencephalographic [32] tests.

Despite its useful functionality, the device shares with other state-of-the-art wearable tactile displays of softness a typical limitation: whilst it allows the contact area to be dynamically varied, it does not make it controllable. This limitation causes indetermination on such an important tactile cue to realistically render the softness of virtual objects.

In general, the contact area may be controlled in a closed loop or open loop. Either strategy requires contact area sensing, which, in principle, could be implemented in two alternative ways: (i) using a dense array of sensors on the membrane to continuously monitor the contact area during operation of the tactile display to enable closed-loop control; (ii) establishing, prior to any usage of the display, a personalised calibration to obtain a curve that relates the contact area to the applied pressure to enable open-loop control.

In either case, the problem is how to make the contact area accurately measurable. In particular, developing a self-sensing elastomeric membrane (for closed-loop control) that allows for monitoring its deformations at a high spatial resolution is challenging. For instance, fabricating dense arrays of stretchable elastomeric capacitors or resistors, serving as miniature tactile elements (taxels), introduces significant complications, especially for routing stretchable electrical connections to read each taxel. Indeed, the connections would occupy space on the membrane, reducing the achievable taxel density and, therefore, also the sensing resolution. As an example, Sonar et al. have described a fingertip-mounted pneumatic chamber with piezo-resistive self-sensing properties, which, however, was limited to single-variable sensing [29].

The challenge of reducing the sensing area to make space for connections in an array of stretchable sensors is currently being tackled through ongoing research into innovative reading strategies. For instance, Xu et al. proposed replacing an array of elastomeric small capacitors with a stack of two elastomeric capacitive membranes, to be virtually partitioned into multiple sensing elements by utilizing a multi-frequency capacitance reading technique, which might eliminate the need for physically addressing each sensing element [33]. However, achieving high resolutions with such a strategy remains difficult, due to the need for resolving decreasing differences in capacitance between adjacent elements as their equivalent size reduces [33].

To overcome such issues implied by sensorisations of the actuation membrane, in this study we developed a strategy and an apparatus to achieve open-loop control of the contact area by means of an optical calibration, as described in the next section.

## 3. Proposed Strategy: Contact Area Open-Loop Control by Personalised Optical Calibration

**Figure 2:**
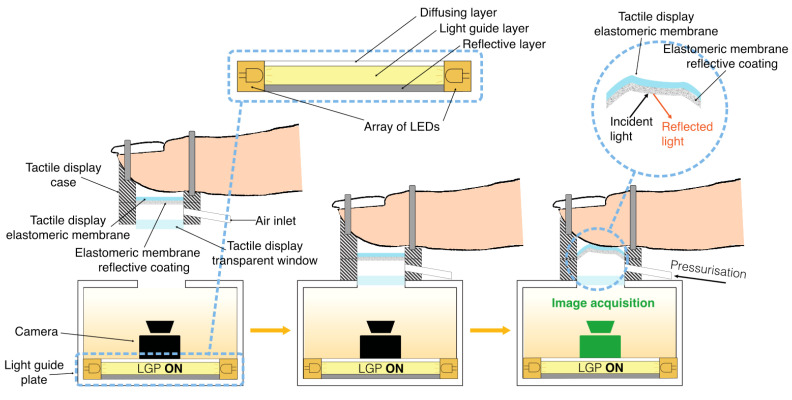
Conceptual schematic representation of the proposed strategy for a personalised optical calibration of the tactile display to enable open-loop control of the contact area: the tactile display’s structure is modified with a rigid transparent window, which allows for optically detecting the contact region as the membrane is inflated against the fingertip. The detection is performed by a camera arranged inside a structure, which acts as an external calibration device, to be used every time that the display is arranged on a finger. The underside of the tactile display’s membrane is coated with reflective particles, which facilitate optical detection of the contact region (figure inset on the right-hand side). Diffused light inside the structure is generated by combining an array of LEDs with a light guide plate, consisting of a planar stack of three layers: a light guide layer, sandwiched between a reflective layer and a diffusing layer (figure inset).

The strategy takes advantage of the general concept of so-called tactile image sensing, which uses cameras to optically monitor deformations occurring in soft membranes when they come into contact with external bodies [34,35,36]. In particular, our strategy envisages that, firstly, the tactile display is modified by creating a rigid transparent window on its lower side, so as to make the internal membrane visible; secondly, the modified display is calibrated with an external device, which integrates a camera and a source of diffused light to illuminate the membrane’s underside; furthermore, to facilitate the identification of the contact surface by the camera, the membrane is coated with reflecting particles. The concept is shown in Figure 2.

The idea is that the finger wearing the tactile display is arranged on top of the external calibration device for a few seconds, so that the internal camera can automatically take a certain number of photos of the variable shape and size of the skin–membrane interface corresponding to a sequence of different applied pressures. A calibration software quantifies the contact area at each pressure to then generate a personalised calibration curve relating those two variables.

This calibration procedure should be repeated every time that the user wears the device. Indeed, the calibration curve is not only finger-specific (according to the fingertip shape and size), but also is expected to vary every time that the tactile display is applied to the same finger (according to a variable arrangement on the fingertip).

In the following sections, we present an implementation of the concept. In particular, we describe the design and assembly of the optical calibration device and a validation of its ability to enable accurate estimates of the contact area via optical measurements by comparing them with measurements obtained using an ink staining method. Moreover, in order to target a fully automated calibration procedure, i.e., an automated determination of the contact area, we describe a performance comparison between a manual process and a preliminary version of an automated routine.

## 4. Materials and Methods

### 4.1. Elastomeric Membrane and Reflective Particles

The tactile display’s elastomeric membrane was optically transparent and consisted of a polydimethylsiloxane (PDMS). It was purchased as a finished product (Elastosil membrane, Wacker, Germany) with a thickness of 70 µm.

The membrane’s underside was coated with a viscous medium loaded with reflective particles, consisting of mineral silicates (mica), commercially available as a cosmetic product (209 silver eyeshadow, Wycon cosmetics, Milan, Italy).

### 4.2. Design and Assembly of the Optical Calibration Device for the Tactile Display

**Figure 3:**
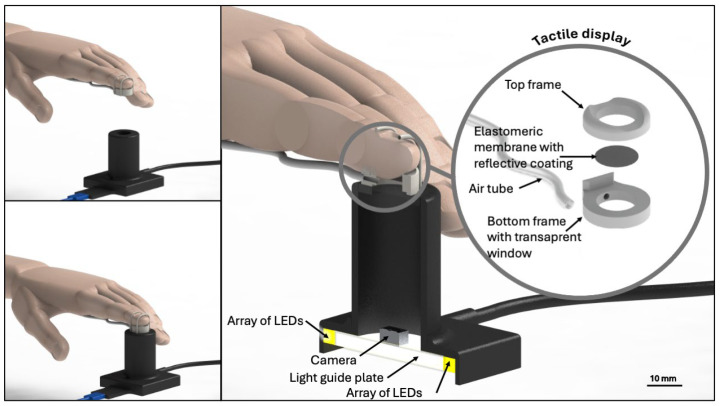
Design of the optical calibration device. The sectional view shows the internal structure. The inset figure presents the constitutive parts of the modified tactile display.

The calibration device consisted of a cylindrical hollow structure made of a 3D-printed plastic material, as shown by the CAD drawings presented in Figure 3.

The base of the structure hosted a camera, which had a resolution of 5 MP (2592 × 1944 pixels) and a focal distance of 60 mm, corresponding to the position of the tactile display’s membrane. To accommodate that distance, the cylinder had a height of 55 mm. Its inner diameter was 27.5 mm, and its edge was shaped to firmly accommodate the tactile display’s case. The latter was re-designed to include a rigid window, consisting of a 2 mm-thick transparent slab of poly(methyl methacrylate), through which the internal elastomeric membrane could be observed by the camera.

Preliminary tests had shown that a direct illumination of the membrane’s reflective particles tended to cause excessive reflection, with a consequent overexposure of the camera, which challenged the contact region detection. That issue was overcome by illuminating the inside of the calibration device with diffused light. The latter was created by combining an array of LEDs with a ring-shaped light guide plate (LGP).

The LGP consisted of a 3 mm-thick slab of transparent acrylic, whose top surface was sanded, and the bottom surface was made reflective by applying aluminium tape, creating the three-layer structure represented in Figure 2. The LGP took light from the LEDs placed along the edge and distributed it internally: the reflective surface at the bottom directed all the incident light towards the top sanded surface, which scattered the exiting light. Furthermore, as a further measure to avoid overexposure of the camera, the internal walls of the structure were made black.

### 4.3. Validation of the Optical Method for Detecting the Contact Area

**Figure 4:**
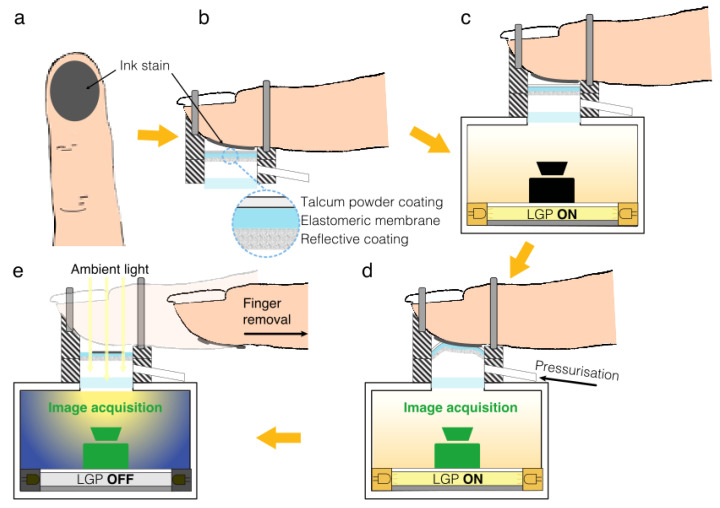
Validation test to investigate the accuracy of the optical method for measuring the contact area: (**a**) coating of the finger pulp with an ink; (**b**) insertion of the finger into the tactile display; (**c**) arrangement of the finger-mounted tactile display on the calibration device; (**d**) application of a test pressure with concomitant imaging of the 3D contact surface, as well as concomitant staining of the membrane by the ink all over the contact surface; (**e**) deflation of the tactile display, causing the stained membrane to flatten, and removal of the finger from the tactile display to let the camera image the stained 2D contact surface.

The strategy and device described above for calibrating the tactile display rely on detecting the contact area by taking photos with an external camera. This implies the use of images created on the 2D plane of the camera sensor by an optical projection of the contact surface, which, however, has a 3D shape. Therefore, it was important to validate that method and assess its accuracy. To that aim, we performed a validation test, using an ink to stain the membrane all over the contact surface to identify and measure it accurately, and make comparisons with estimates provided by the optical method. The validation test was performed as described below, with reference to Figure 4.

As a first step, the finger pulp was entirely coated with a water-based ink (Figure 4a). In consideration of the fact that the ink was incompatible with the hydrophobicity of the silicone-made elastomeric membrane, the latter was coated with talcum powder; this allowed the ink to be smoothly transferred from the finger to the membrane.

Then, the finger was inserted into the tactile display (Figure 4b) and was arranged on the calibration device (Figure 4c). With the internal diffused light turned on, the camera took a picture of the 3D contact surface corresponding to a given pressurisation of the tactile display (Figure 4d), according to the optical method described above. However, at this stage, the membrane also became stained by the ink all over the contact surface.

Subsequently, the pressure was brought back to zero, so that the membrane lost contact with the finger, becoming flat; at this stage, the finger was removed from the tactile display, and the internal light was turned off. Upon illuminating the flattened membrane with external light, the camera was used to image the stain left on the membrane by the ink, corresponding to the 2D contact surface (Figure 4e).

The whole process was repeated for different pressures, which were presented in ascending order, to ensure that the stain was growing at each step.

It is worth noting that, while the 3D contact surface was revealed by the reflecting particles on the membrane’s side facing the camera, the 2D contact surface was revealed by the ink on the membrane’s other side. The identification of the ink stain was enabled by the membrane’s transparency and was not significantly limited by the presence of the reflective particles, as the internal light was turned off.

Imaging the stain when the membrane was brought back to a flat shape ensured that the contact area was estimated with the highest possible accuracy. Indeed, the membrane’s 2D spatial arrangement did not alter the optical projection of the contact surface on the camera sensor’s 2D plane. This enabled validation of the accuracy of estimating the contact area when the membrane is deformed by contact with the finger, as envisaged by the optical method.

For the implementation of this test, each contact surface was identified ‘manually’: each photo taken at each pressure was visually inspected to distinguish the edges of the contact region and manually mark them (as detailed below). This procedure ensured the highest possible accuracy to quantify the contact area, as required for the validation. Nevertheless, whilst such a manual procedure was applicable to this validation test (which had to be performed only once), clearly it would not be usable during the actual calibration process. The latter would require an automated procedure. Therefore, an image processing algorithm for quantifying the contact area was also developed, as described in the following.

### 4.4. Manual Procedure for Quantifying the Contact Area

To obtain the reference values of the contact area, we implemented a manual procedure, which envisaged an analysis of the photos with the software ImageJ. Its tool ‘*Elliptical*’ was used to select the contact region by adapting a variable ellipse around its edges. To that end, the two axes of the ellipse were independently modified, until a satisfactory geometrical fitting was achieved. The software returned the area of the ellipse in pixels, which was then converted into square millimetres.

In order to overcome the limitations that such a manual procedure would introduce into the calibration process (in terms of ease, speed and reliability), we also developed an algorithm to detect the contact area automatically, as detailed below.

### 4.5. Development and Evaluation of a Preliminary Procedure for Quantifying the Contact Area Automatically

A preliminary automated procedure was developed using the programming language Python and the computer vision library OpenCV. The algorithm was conceived to accomplish two main consecutive tasks: (i) identifying the region of interest (ROI), defined as the circular portion of the image where the contact region was likely to be found; (ii) identifying the contact region within the ROI and quantifying the contact area.

**Figure 5:**
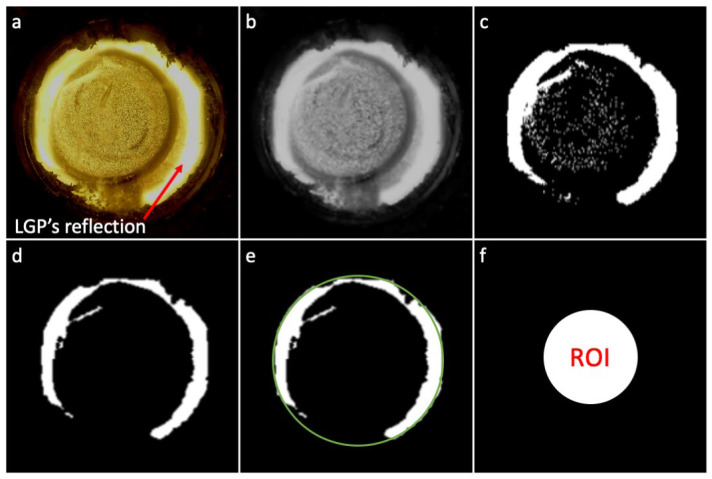
Automated procedure for identifying the circular ROI: (**a**) Example of an image captured by the camera; note that the peripheral ring with the highest brightness was a reflection (on the tactile display’s transparent window) of the ring-shaped LGP. (**b**) The image was converted from the RGB format to the grayscale format. (**c**) A binary thresholding was applied to the grayscale image. (**d**) To remove noise, the image was eroded. (**e**) The image was segmented and the contour with the largest area was assumed to correspond to the reflection of the ring-shaped LGP; the contour was fitted with the smallest area circle. (**f**) According to empirical evidence, a suitable circular ROI was defined by scaling the circle diameter by a factor of 55%.

The identification of the ROI was performed on the image corresponding to null pressure, according to the procedure described below, with reference to Figure 5.

The image (Figure 5a) was initially converted from the RGB format to the grayscale format using the OpenCV function ‘cvtColor’ (Figure 5b). A binary thresholding (using the OpenCV function ‘threshold’ with a brightness threshold of 150) was applied to the grayscale image, assigning the minimum possible value of brightness (0) to all the pixels below the threshold, and the maximum possible value of brightness (255) to all the pixels above the threshold, obtaining a binary image (Figure 5c). To remove the noise represented by the small spots of brightness visible in the binary image, the latter was eroded, using the OpenCV function ‘erode’, with a circular kernel of 10 pixels in diameter, obtaining a new binary image (Figure 5d). To identify connected zones, the image was then segmented, using the OpenCV function ‘findContours’; among the connected contours returned by the function, the one with the largest area was assumed to correspond to the reflection of the ring-shaped LGP. That contour was then fitted with the smallest area circle using the OpenCV function ‘minEnclosingCircle’, obtaining the smallest area circle that completely covered the contour (Figure 5e). That circle was used to identify the circular ROI as a concentric circle with a smaller diameter; in particular, according to the adopted setup and experimental conditions (including the image magnification and the size of the LGP reflection ring appearing on the image—Figure 5a), we empirically found that a suitable ROI was given by a 55% scaling of the diameter (Figure 5f).

**Figure 6:**
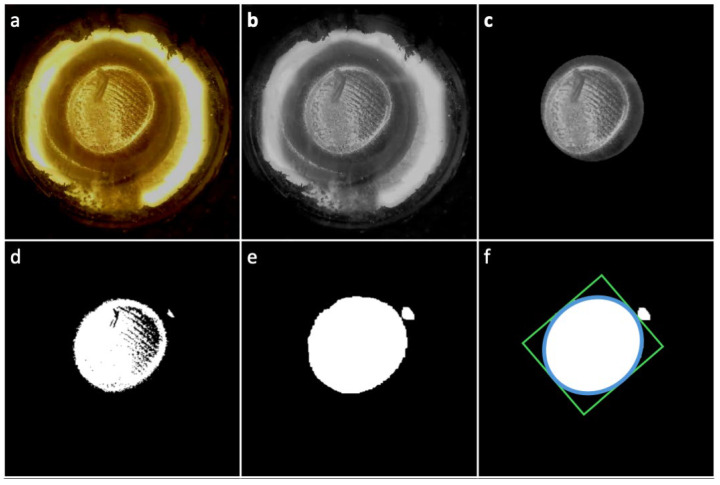
Automated procedure for identifying the contact region within the ROI and quantifying the contact area: (**a**) Example of an image captured by the camera. (**b**) The image was converted from the RGB format to the grayscale format. (**c**) The grayscale image was masked, according to the ROI. (**d**) The masked image was converted into a binary image. (**e**) To remove noise, the image was eroded, and to enhance object boundaries, fill small gaps and connect nearby objects, it was also dilated. (**f**) The image was segmented and the contour with the largest area was assumed to correspond to the contact region; its area was calculated by adapting a rectangle and considering the area of an inscribed tangent ellipse.

The identification of the contact region within the ROI and the quantification of the contact area were performed on each image at any given pressure, according to the procedure described below, with reference to Figure 6.

The image (Figure 6a) was converted from the RGB format to the grayscale format using the OpenCV function ‘cvtColor’ (Figure 6b). Then, the circular ROI (identified in the previous step) was used to mask the image by computing a pixel-to-pixel logical ‘and’, using the NumPy function ‘bitwise_and’ (Figure 6c). The masked grayscale image was converted into a binary image by means of binary thresholding (using the OpenCV function ‘threshold’ with a threshold equal to half of the image’s maximum brightness), which applied the values 0 to all the pixels below the threshold and 255 to all the pixels above the threshold (Figure 6d). Note that, unlike the thresholding operation performed to determine the ROI, in this phase the threshold did not have a predefined value; this was due to the fact that different images taken at different pressures could exhibit different values of brightness because of a different reflection of light caused by a different curvature of the membrane in contact with the finger. Then, to remove noise, the image was eroded, using the OpenCV function ‘erode’ with a circular kernel of 3 pixels in diameter; subsequently, to enhance object boundaries, fill small gaps and connect nearby objects, the image was dilated using the OpenCV function ‘dilate’ with a circular kernel of 15 pixels in diameter. As a result, the new image shown in Figure 6e was obtained. To identify connected zones, the image was then segmented using the OpenCV function ‘findContours’; among the connected contours returned by the function, the one with the largest area was assumed to correspond to the contact region. The minimum area rectangle that bounded the contour (highlighted in green in Figure 6f) was identified using the OpenCV function ‘minAreaRect’, which provided the lengths of the two sides of the rectangle. Those values were used to calculate the contact area as the area of the rectangle-fitting ellipse (highlighted in light blue in Figure 6f), i.e., the ellipse whose two axes had the same lengths as the sides of the rectangle.

This procedure was repeated for each image at a different pressure.

## 5. Results and Discussion

### 5.1. Optical Calibration Device

**Figure 7:**
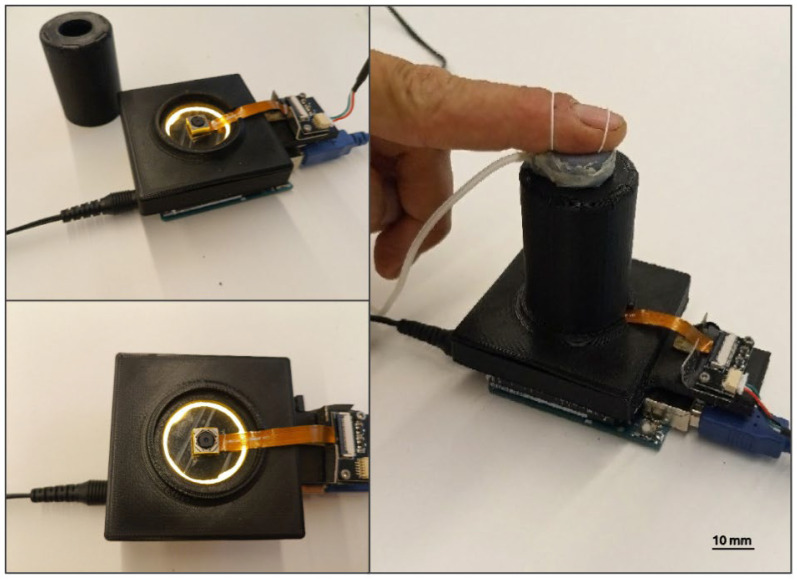
Implementation of the optical calibration device. The left-hand side photos show the internal camera and LGP. The latter is visible as a ring of light; its central part was black because it was masked (with a black tape) to avoid excessive illumination of the elastomeric membrane, which would have caused excessive reflection by the particles on its surface, leading to overexposure of the camera.

A prototype implementation of the optical calibration device is shown in Figure 7.

This device was used to validate the accuracy of the optical strategy for detecting the contact area, as well as the performance of an algorithm for its quantification in an automatic way, as separately reported in the following sections.

### 5.2. Validation of the Optical Method for Detecting the Contact Area

The validation test of the optical calibration method was aimed at assessing its accuracy through a comparison between a set of contact area measurements taken when the membrane was deformed (Figure 4d), as envisaged by the calibration method, and a set of corresponding measurements (having the highest possible accuracy) taken when the stained membrane had become flat again (Figure 4e).

**Figure 8:**
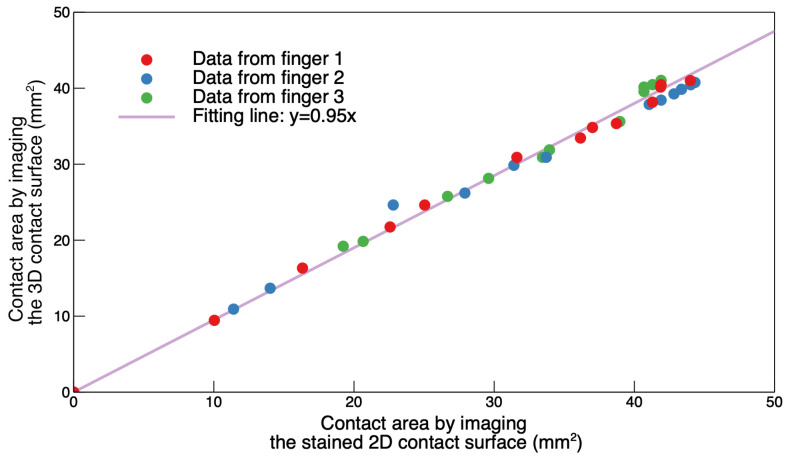
Validation of the optical method for detecting the contact area. The area measured when the membrane was in contact with the finger upon pressurisation is plotted as a function of the corresponding area measured when the membrane had become flat again after depressurisation. The data points refer to measurements taken from three fingers, within the pressure range 0–20 kPa. Note that the contact area ranges for the three fingers were different, as the fingers had different sizes and shapes.

The contact region was identified by manually processing the images, according to the same methodology described in the next section (where it is compared with the preliminary version of an automated procedure). The results of the validation test are presented in Figure 8.

The linearity shown by the graph indicates that the optical method enabled reliable detections of the contact area.

### 5.3. Evaluation of the Preliminary Procedure for Quantifying the Contact Area Automatically

In order to assess the accuracy of the preliminary procedure that was developed for quantifying the contact area automatically, we compared a set of contact area measurements taken manually and a set of corresponding measurements returned by the procedure.

**Figure 9:**
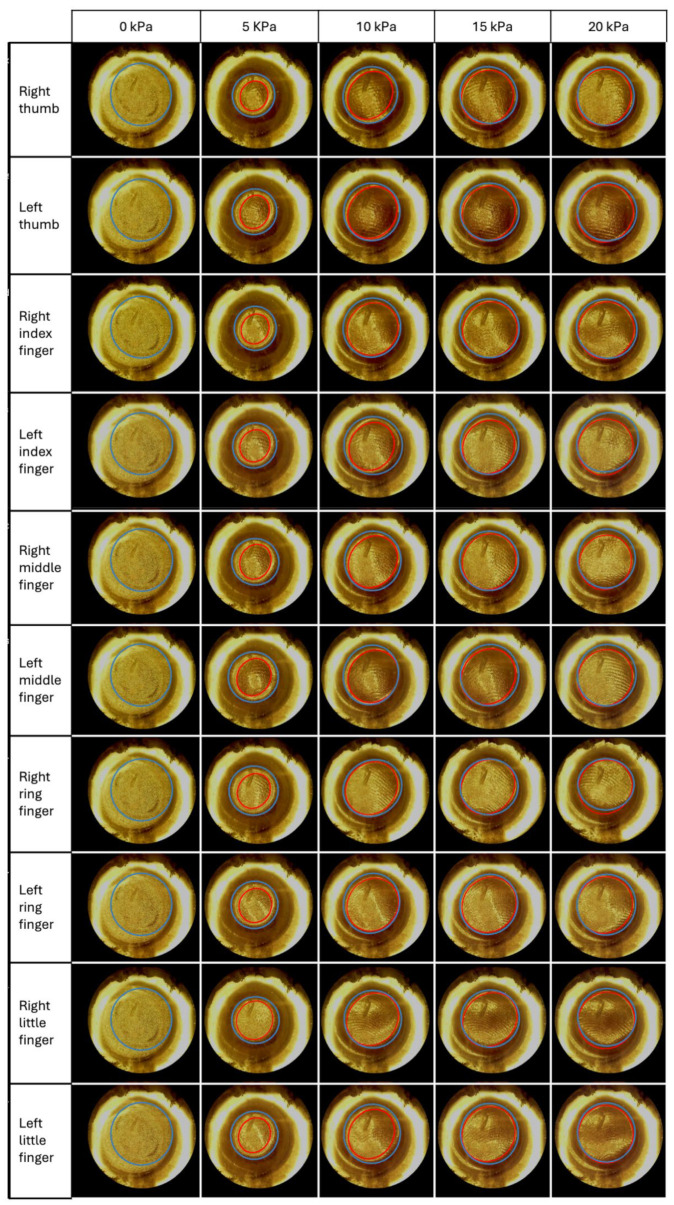
Evaluation of the ability of the automated image-processing algorithm to identify the contact region, relative to the manual identification. The region identified with the manual method (red ellipse) is compared to that returned by the automated procedure (blue ellipse). The comparisons were made on ten fingers, within the pressure range 0–20 kPa. For simplicity, here we present only a few sample images (i.e., those at 0, 5, 10, 15 and 20 kPa). Note that the automated procedure’s estimate of the contact region at null pressure was meaningless, as discussed below.

Figure 9 presents such a comparison, showing the differences in the contact region achieved with the two methods.

All the images (except for those at null pressure, which are discussed later on) show that the automated procedure was able to identify the contact region rather well. However, the procedure also tended to systematically generate overestimates.

**Figure 10:**
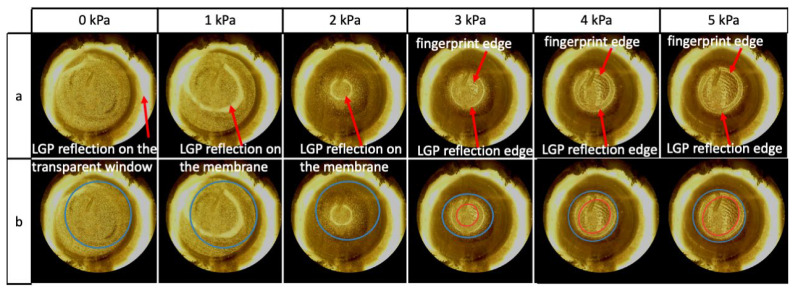
Inability of the automated image processing algorithm to identify the contact region at low pressures. The figure shows for one finger pulp, taken as an example, a sequence of images at 0, 1, 2, 3, 4 and 5 kPa. The original images (**a**) are repeated in (**b**) with the overlapping ellipses used in the manual procedure (red curve) and automated procedure (blue curve). Up to ~3 kPa, the automated procedure returned meaningless estimates.

Moreover, the automated procedure was found to be unable to provide meaningful outcomes at low pressures. In order to clarify this aspect, Figure 10 presents, as an example, a set of images taken from a single finger pulp at 0, 1, 2, 3, 4 and 5 kPa.

From 3 kPa onwards, the visualisation of the fingerprints was indicative of an established contact between the membrane and the finger pulp; at lower pressures, the lack of fingerprints revealed that contact was either absent or too weak (Figure 10).

The manual definition of the contact region was performed as described below.

First, it is worth noting that each image showed not only a peripheral ring of light, corresponding to a reflection of the LGP on the tactile display’s transparent window (as recalled above), but also an internal luminous ring (Figure 10). The latter was generated by a reflection of the LGP on the curved membrane. Indeed, it was absent in the image at 0 kPa (as the membrane was flat) and varied its size and shape with increasing pressures, according to the membrane’s inflation and the geometry of the region of contact with the finger. In particular, the internal ring decreased its size from 1 to 2 kPa because the membrane was inflated, while remaining detached from the finger. Then, at 3 kPa, the membrane had established contact with the finger pulp (as indicated by the fingerprints), causing an enlargement of the reflection ring. Such an enlargement progressively continued with increasing pressures, as the contact region spread (Figure 10).

Furthermore, it is important to observe that, with increasing pressures, the growing fingerprint region was delimited by an inner ring with an increasingly sharp edge (Figure 10). We therefore considered that inner edge as a reliable marker of the contact region.

**Figure 11:**
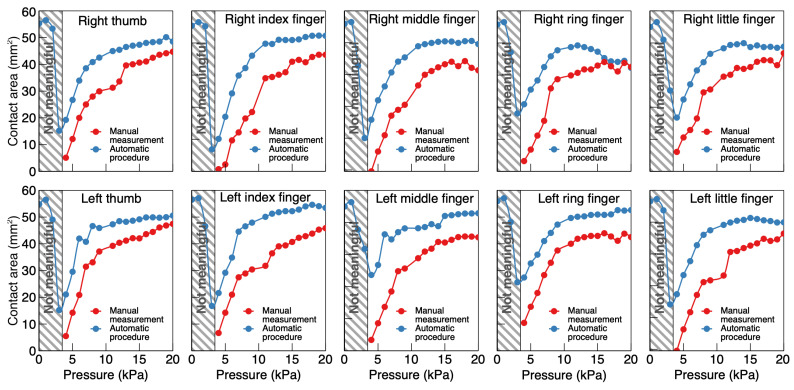
Evaluation of the ability of the automated image-processing algorithm to quantify the contact area, relative to the manual quantification. The areas quantified with the manual method (red dots) are compared to those returned by the automated procedure (blue dots). The comparisons were made on ten fingers, within the pressure range 0–20 kPa. Note that for all fingers used in this test, the automated procedure returned meaningless estimates up to ~3 kPa; up to that pressure, the tactile display’s membrane likely did not come into contact with the finger pulp (the images did not show any visible fingerprint) and, so, there was no contact region to be identified.

By using that marker, the contact area was manually determined at each pressure and compared with the area returned by the automatic procedure. The results are presented in Figure 11.

**Figure 12:**
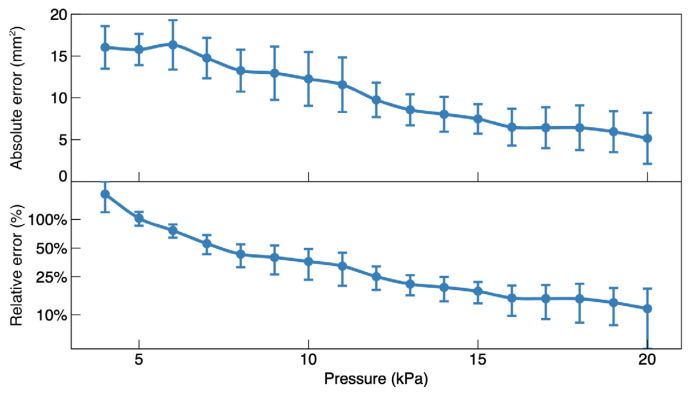
Absolute and relative errors made in the automated estimate of the contact area. The error bars refer to the standard deviation among 10 fingers.

The error made by the automated procedure (relative to the manual process) in overestimating the contact area at each pressure is plotted in Figure 12, both in absolute and relative terms.

While the relative error was significantly larger at low pressures, it quickly decreased for increasing pressures, reaching the order of 10% at the highest end of the pressure range. Although a 10% error might be satisfying for a diversity of possible applications, the higher errors at low pressures would prevent reliable uses of such an automated procedure within the whole working range of the device.

This limitation requires further developments, aimed at improving the accuracy of automated estimates of the contact area. For instance, this issue might be addressed with deep learning approaches, using artificial neural networks, to be trained with a diversity of images of different contact regions.

## 6. Conclusions and Future Developments

This study addressed the problem of controlling the contact area between a fingertip and the soft membrane of a pneumatic tactile display of softness to better render the softness of virtual objects.

The proposed approach was based on the development of a strategy to achieve a calibration that related the contact area to the driving pressure (enabling open-loop controls), and to make that calibration personalised. A personalisation was regarded as necessary to account for the variability in size and shape across different fingers, as well as a possible variability even in the way that the device is arranged on any given finger at different times.

The proposed strategy consisted of using an external device to obtain such a calibration via an optical method, which proved effective.

Although the method here was developed for a tactile display that was actuated pneumatically, it may also be applied to other kinds of devices, which might have different actuation technologies, while still establishing contact with the skin via transparent soft membranes.

Advantageously, estimating (at any given pressure) the contact area using such a calibration is significantly simpler, and possibly even more accurate, than conceivable alternatives based on embedding dense arrays of miniature tactile sensors within the membrane (as discussed in the Introduction). Indeed, the optical strategy uses just a single sensor (a camera), which can detect the contact area without any special elastomeric membrane, and also at higher resolutions.

In our tests, the accuracy in measuring the contact area was largely limited by two main factors: (i) the quality of the captured images; (ii) the procedure adopted to identify the contact region, ideally in an automatic way.

The image quality depended on several aspects, including the conditions of illumination and contrast, as well as the presence of undesired reflections from internal parts (e.g., the LGP, as shown in Figure 10); this requires future improvements on the optical design of the calibration unit.

The procedure to identify the contact region should be improved (to increase the accuracy of identification of the edges) and should be automated, avoiding manual processes (to make the operation faster, easier and more reliable); this requires future developments of the described preliminary routine, which may also be replaced with more effective strategies, possibly based on deep learning strategies.

Nevertheless, it is worth noting that the accuracy required to control the contact area is currently unknown. Indeed, while the tactile resolution in finger pulps is known to be as low as ~0.3 mm (tactile hyperacuity) [37], there is no evidence that controlling the contact area for rendering a virtual softness requires an accuracy so high. Therefore, future developments should, firstly, investigate the perceptual requirements and, secondly, seek for an acceptable trade-off between those requirements and possible technological limitations.

Once the system is optimised, it may be used to investigate whether making the contact area controllable can ensure that the perceptual experience of exploring the softness of a virtual object is improved to a significant extent. In particular, it will be important to assess (with psychophysical tests) whether any improvement will be so significant as to be worthy of the initial calibration process prior to any usage.

## Data Availability

The data presented in this study are available on request from the corresponding author.
